# Patient characteristics, antibiotic use, and in‐hospital outcomes in patients with ischaemic colitis: A nationwide retrospective cohort study

**DOI:** 10.1111/codi.70385

**Published:** 2026-02-08

**Authors:** Yasuhiro Kano, Yuya Kimura, Hiroki Matsui, Kiyohide Fushimi, Hideo Yasunaga

**Affiliations:** ^1^ Department of Clinical Epidemiology and Health Economics, School of Public Health University of Tokyo Tokyo Japan; ^2^ Department of Emergency and General Medicine Tokyo Metropolitan Tama Medical Center Tokyo Japan; ^3^ Department of Health Services Research, Graduate School of Medicine University of Tokyo Tokyo Japan; ^4^ Department of Health Policy and Informatics Institute of Science Tokyo Graduate School of Medical and Dental Sciences Tokyo Japan

**Keywords:** anti‐bacterial agents, colitis, ischaemic, Japan, retrospective studies

## Abstract

**Aim:**

To describe patient characteristics, management and in‐hospital outcomes of ischaemic colitis, and to compare the rates of surgery and in‐hospital death between patients who did and did not receive antibiotics.

**Method:**

We retrospectively identified hospital admissions for ischaemic colitis between April 2016 and March 2023 from the Diagnosis Procedure Combination inpatient database in Japan. We described the overall patient characteristics, management practices and outcomes, which were stratified by antibiotic use. The primary outcome was a composite of receipt of surgery and in‐hospital mortality. We examined the association between antibiotic use within the first 2 days of admission and the primary outcome occurring on or after day 3 of hospitalization using multivariable logistic regression analyses.

**Results:**

Among 111,750 eligible cases, 36.2% received antibiotics within the first 2 days of admission. Of them, 0.9% underwent surgery and 1.3% died during hospitalization. The primary outcome occurred in 2.1% of cases in the overall cohort (≤2 days: 0.6%; ≥3 days: 1.5%) and in 1.0% of cases who did not receive (≤2 days: 0.2%; ≥3 days: 0.8%) and 4.2% of cases who received antibiotics (≤2 days: 1.5%; ≥3 days: 2.7%). After covariate adjustment, antibiotic use was associated with higher odds of the primary composite outcome (adjusted odds ratio 1.98, 95% confidence interval: 1.77–2.21).

**Conclusion:**

The surgical rate and in‐hospital mortality in ischaemic colitis were lower than previously reported. Antibiotic use was associated with higher odds of surgery and in‐hospital mortality; however, this finding should be interpreted cautiously, given potential unmeasured confounding.

**What does this paper add to the literature?**

Epidemiological data on outcomes and the effectiveness of antibiotics in ischaemic colitis are limited. In this nationwide study, the surgical rate (0.9%) and in‐hospital mortality (1.3%) in ischaemic colitis were lower than previously reported. About one‐third of patients received antibiotics, with higher surgery and mortality rates, implying severe disease.

## INTRODUCTION

The incidence of ischaemic colitis has risen worldwide [1, 2]. The age‐ and sex‐adjusted incidence rates rose from 6.1 cases per 100,000 person‐years in 1976–1980 to 22.9 per 100,000 in 2005–2009 [2]. The incidence is expected to increase due to the ageing population, as advancing age and multimorbidity are risk factors for ischaemic colitis [3]. However, real‐world evidence on patient characteristics, current management practices and outcomes is limited because most prior studies enrolled small samples or focused on specific populations, such as patients who underwent surgery or those with histologically confirmed ischaemic colitis [4]. Consequently, reliable epidemiological data are scarce.

The American College of Gastroenterology (ACG) guideline strongly recommends broad‐spectrum antimicrobials as part of conservative management for moderate‐to‐severe disease [5]. In the guideline, disease severity is classified based on the number of risk factors (including sex, vital signs, laboratory findings and colonoscopy findings) and the presence of findings suggestive of peritonitis or bowel necrosis on physical examination, imaging or colonoscopy. Patients with ischaemic colitis are often prescribed antibiotics in a bid to minimize bacterial translocation and sepsis and reduce the severity and extent of bowel damage [6]. However, these rationales have been derived largely from studies on animal models rather than human data [6, 7]. Recent small observational studies found no association between antibiotic use and death, surgery or readmission, while reporting longer hospital stays among patients receiving antibiotics [7, 8]. To date, no large‐scale study has evaluated the association between antibiotic use and outcomes in ischaemic colitis [9, 10]. Because a substantial proportion of patients with ischaemic colitis are prescribed antibiotics [11], this issue is important not only for improving the quality of treatment of ischaemic colitis but also for antimicrobial stewardship [12].

To address these knowledge gaps, large‐scale, real‐world data are needed to quantitatively describe the patient characteristics, real‐world practice and outcomes of ischaemic colitis. Therefore, we conducted a retrospective cohort study using a nationwide inpatient database in Japan. Furthermore, we aimed to compare the rates of surgery and in‐hospital death between patients who did and did not receive antibiotics.

## METHODS

### Study design and data source

This retrospective cohort study analysed data extracted from the Japanese Diagnosis Procedure Combination (DPC), a nationwide inpatient database encompassing 1,786 acute‐care hospitals in Japan [13]. The database includes hospital identifiers, patient demographics, diagnoses (including the main diagnosis, admission‐precipitating diagnosis, comorbidities present at admission and complications arising after admission), admission and discharge status, surgical procedures and administrative claims data, including medication prescriptions. The diagnoses are recorded as International Classification of Diseases–10th Revision (ICD‐10) codes, Japanese standard disease codes and text data. Since one ICD‐10 code may encompass multiple conditions, combining ICD‐10 codes with Japanese standard disease codes or text data ensures a more precise case definition. The accuracy of the diagnostic and procedural records in the DPC has been validated to be generally high [13, 14]. This study was approved by the Institutional Review Board of the University of Tokyo (3501–5, May 19, 2021), which waived the need for informed consent due to the anonymous nature of the data source.

### Patient selection

We initially identified candidate cases of ischaemic colitis recorded using ICD‐10 codes K55.0 or K55.9 as the main or admission‐precipitating diagnosis between April 1, 2016, and March 31, 2023 (Japanese fiscal years 2016–2022). Subsequently, we selected cases with Japanese standard disease codes designating ‘acute ischaemic enteritis’ (8851056), ‘acute ischaemic colitis’ (8845883), ‘ischaemic enteritis’ (5579002) or ‘ischaemic colitis’ (5579004) as the main or admission‐precipitating diagnosis. Suspected diagnoses were not included. We excluded patients aged <18 years. To improve diagnostic specificity, we also excluded patients without computed tomography (CT) scans acquired within 2 days of admission. Patients with gastrointestinal perforation or intra‐abdominal abscess on admission were excluded because these conditions are absolute indications for surgery and/or antibiotic therapy and are therefore antithetical to our study aims. Repeat hospitalizations for ischaemic colitis in the same patient during the study period were included as separate cases.

### Data for descriptive analysis

We clarified the following patient characteristics: age, sex, body mass index (BMI), smoking history, Barthel Index, Charlson Comorbidity Index, comorbidities at admission (defined by ICD‐10 codes; Supplemental Digital Content Table S1), intensive care unit (ICU) or high‐dependency care unit (HDU) admission, admission to a teaching hospital, attending department and fiscal year.

We also described the clinical practice patterns, including rates of antibiotic use, rates and duration of intravenous fluid therapy, rate of receiving surgery and distribution of surgery type, rate and duration of fasting, rate for colonoscopy and volume of red blood cell transfusion during hospitalization. All commonly used oral and intravenous antibiotics in Japan were collated. However, oral macrolides and sulphonamides were excluded because the DPC database captures only in‐hospital medication use and does not contain pre‐admission medication histories, which prevented us from identifying long‐term maintenance use. Intravenous fluid therapy was defined as the administration of a preparation >100 mL in a single dose, to distinguish between therapeutic hydration and solvent use because 50–100 mL solutions are commonly used as diluents in Japan. The descriptions of the intravenous fluid therapy agents are provided in Supplemental Digital Content Table S2. Treatments and interventions were attributed to ischaemic colitis management only if initiated within the first 2 days of hospitalization, to reduce misclassification due to other indications.

The primary outcome was a composite of surgery and in‐hospital mortality during the entire hospitalization. This outcome was chosen because preventing the need for surgery resulting from bowel injury and preventing in‐hospital death potentially related to bacterial translocation and sepsis are key intended clinical goals of antibiotic use in ischaemic colitis. The secondary outcomes included the occurrence of *Clostridioides difficile* infection (defined as an ICD‐10 code of A04.7), length of hospital stay, 30‐day readmission for any cause and total hospital costs during admission. Total hospital costs were obtained from the DPC database and represent estimates calculated using Japan's national fee schedule reference prices for itemized inpatient services (e.g. procedures, medications, laboratory tests and other inpatient services) [13].

### Statistical analysis

Continuous variables were summarized as medians with interquartile ranges, and categorical variables as frequencies and percentages. We conducted univariate and multivariate logistic regression exploratory analyses to evaluate the associations between antibiotic use (initiated within 2 days of admission) and the primary outcome (a composite of the need for surgery and in‐hospital mortality). The following covariates were selected a priori in reference to prior studies and presumed association with the outcome based on clinical judgement [2, 9, 10, 15, 16, 17]: age, sex, smoking status, BMI, Barthel Index, Charlson Comorbidity Index, hypertension, dyslipidaemia, diabetes mellitus, atrial fibrillation, heart failure, coronary artery disease, cerebrovascular disease, peripheral artery disease, chronic obstructive pulmonary disease, chronic kidney disease, chronic liver disease, haematological malignancy, solid cancer, connective tissue disease, constipation, fiscal year of admission, admitting department, ICU admission, HDU admission, teaching hospital status, bowel rest at admission, colonoscopy at admission, red blood cell transfusion at admission and intravenous fluid administration at admission. Missing data were categorized as ‘missing’. To avoid immortal time bias and reverse causality, we limited the exploratory analyses to patients who were not discharged (alive or deceased) within 2 days of admission and did not undergo surgery during this period. We reported the odds ratios (ORs) with 95% confidence intervals (CIs). A two‐sided *p* value <0.05 was considered statistically significant. All statistical analyses were performed using R, version 4.4.3 (R Foundation for Statistical Computing, Vienna, Austria). For sensitivity analysis, we reanalysed the data after excluding records of recurrent hospitalizations for ischaemic colitis (i.e. analyses restricted to first admissions for ischaemic colitis).

## RESULTS

### Patient characteristics

**FIGURE 1:**
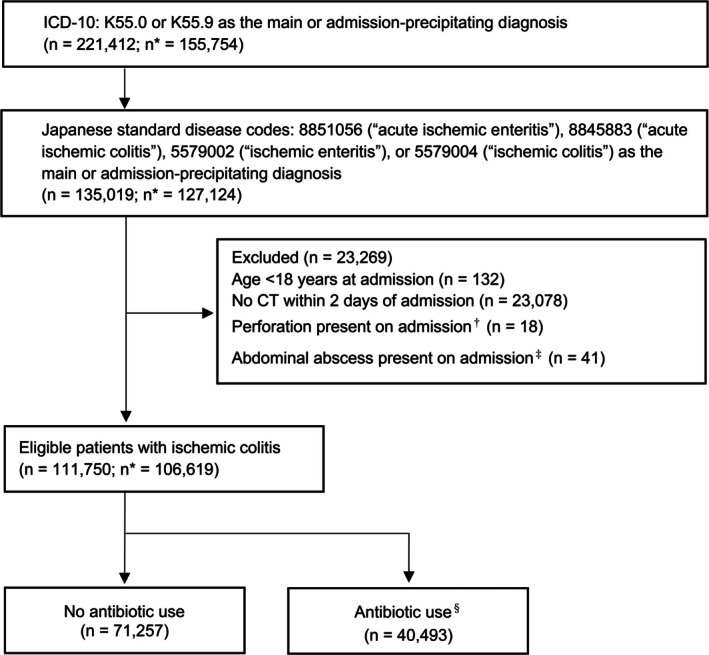
Study flowchart. CT, computed tomography; ICD‐10, International Classification of Diseases–10th Revision. *Numbers of distinct patients. ^†^Defined as an ICD‐10 code of K57.0, K57.2 or K57.8 among the comorbidities present on admission. ^‡^Defined as a standard disease code for perforation (5672002, 8831491, 8833671, 8842723, 8850425, 8836093, 8837056, 8837410, 8837701, 8837718, 8842521, 8839645, 8839650, 8839653 or 8840590) or an ICD‐10 code for abdominal abscess (T81.4), among the comorbidities present on admission. ^§^Antibiotic use for ischaemic colitis was defined as the initiation of antibiotics within the first 2 days of hospital admission.

The overall cohort included 111,750 cases hospitalized for ischaemic colitis from 106,619 distinct patients during the study period (Figure 1). Table 1 presents the baseline patient characteristics in the overall cohort and those stratified by antibiotic use within the first 2 days of admission. The median [interquartile range (IQR)] age of the overall cohort was 74 [63–83] years, and 73.7% of patients were women. Compared with the no‐antibiotic group, patients in the antibiotic group were older, had poorer functional status (i.e. lower Barthel Index scores) and had greater comorbidities. Admissions to the HDU and ICU, as well as admissions under surgical services, were more frequent in the antibiotic group. Compared to the gastrointestinal, general medicine and general internal medicine services, surgical services tended to manage older patients (median age: 75 vs. 74 years), patients with a poorer functional status (Barthel Index: 0: 9.6% vs. 6.9%), patients with a higher comorbidity burden (Charlson Comorbidity Index ≥3: 6.3% vs. 3.7%) and patients admitted to the HDU/ICU (Supplemental Digital Content Table S3).

**TABLE 1:** Patient characteristics stratified by antibiotic use.

Variables	Total (*n* = 111750)	No antibiotic use (*n* = 71257)	Antibiotic use^a^ (*n* = 40493)	SMD
Age, years	74.0 [63.0, 83.0]	73.0 [61.0, 82.0]	77.0 [66.0, 84.0]	0.22
Female	82304 (73.7)	53722 (75.4)	28582 (70.6)	0.11
BMI, kg/m^2^				
	81819 (73.2)	52417 (73.6)	29402 (72.6)	0.02
≥25	21659 (19.4)	13961 (19.6)	7698 (19.0)	0.01
Missing data	8272 (7.4)	4879 (6.8)	3393 (8.4)	0.06
Smoking history				
Non‐smoker	81146 (72.6)	52294 (73.4)	28852 (71.3)	0.05
Current or former smoker	19886 (17.8)	12567 (17.6)	7319 (18.1)	0.01
Missing data	10718 (9.6)	6396 (9.0)	4322 (10.7)	0.06
Barthel Index on admission				
100	66166 (59.2)	45367 (63.7)	20799 (51.4)	0.25
55–95	22529 (20.2)	14316 (20.1)	8213 (20.3)	0.00
5–50	14440 (12.9)	7724 (10.8)	6716 (16.6)	0.17
0	8487 (7.6)	3763 (5.3)	4724 (11.7)	0.23
Missing data	128 (0.1)	87 (0.1)	41 (0.1)	0.01
Charlson Comorbidity Index				
0	84867 (75.9)	56111 (78.7)	28756 (71.0)	0.18
1	7119 (6.4)	4104 (5.8)	3015 (7.4)	0.07
2	14984 (13.4)	8519 (12.0)	6465 (16.0)	0.12
≥3	4780 (4.3)	2523 (3.5)	2257 (5.6)	0.10
Comorbidities				
Hypertension	31359 (28.1)	19224 (27.0)	12135 (30.0)	0.07
Dyslipidaemia	15765 (14.1)	10071 (14.1)	5694 (14.1)	0.00
Diabetes mellitus	14211 (12.7)	8016 (11.2)	6195 (15.3)	0.12
Atrial fibrillation	2871 (2.6)	1670 (2.3)	1201 (3.0)	0.04
Congestive heart failure	4674 (4.2)	2580 (3.6)	2094 (5.2)	0.08
Ischaemic heart disease	7876 (7.0)	4675 (6.6)	3201 (7.9)	0.05
Cerebrovascular disease	7110 (6.4)	4059 (5.7)	3051 (7.5)	0.07
Peripheral artery disease	498 (0.4)	240 (0.3)	258 (0.6)	0.04
COPD	852 (0.8)	466 (0.7)	386 (1.0)	0.03
Chronic kidney disease	3608 (3.2)	1905 (2.7)	1703 (4.2)	0.08
Chronic liver disease	1692 (1.5)	1039 (1.5)	653 (1.6)	0.01
Hematologic malignancy	393 (0.4)	229 (0.3)	164 (0.4)	0.01
Solid cancer	13627 (12.2)	8454 (11.9)	5173 (12.8)	0.03
Connective tissue disease	905 (0.8)	522 (0.7)	383 (0.9)	0.02
Constipation	13941 (12.5)	8956 (12.6)	4985 (12.3)	0.01
HDU admission^b^	761 (0.7)	250 (0.4)	511 (1.3)	0.10
ICU admission^b^	423 (0.4)	61 (0.1)	362 (0.9)	0.12
Teaching hospital admission	91109 (81.5)	59178 (83.0)	31931 (78.9)	0.11
Attending department				
Gastroenterology	57815 (51.7)	38606 (54.2)	19209 (47.4)	0.14
Surgery	13907 (12.4)	6712 (9.4)	7195 (17.8)	0.25
General (Internal) Medicine	34730 (31.1)	22914 (32.2)	11816 (29.2)	0.06
Other	5298 (4.7)	3025 (4.2)	2273 (5.6)	0.06
Fiscal year				
2016	16514 (14.8)	10069 (14.1)	6445 (15.9)	0.05
2017	16417 (14.7)	10149 (14.2)	6268 (15.5)	0.03
2018	17121 (15.3)	10794 (15.1)	6327 (15.6)	0.01
2019	16351 (14.6)	10797 (15.2)	5554 (13.7)	0.04
2020	16883 (15.1)	10886 (15.3)	5997 (14.8)	0.01
2021	15314 (13.7)	9928 (13.9)	5386 (13.3)	0.02
2022	13150 (11.8)	8634 (12.1)	4516 (11.2)	0.03

*Note*: Continuous variables are presented as the median [IQR], and categorical variables as *n* (%).

Abbreviations: BMI, body mass index; COPD, chronic obstructive pulmonary disease; HDU, high‐dependency care unit; ICU, intensive care unit; IQR, interquartile range; SD, standard deviation; SMD, standardized mean difference.

^a^ Antibiotic use for ischaemic colitis was defined as the initiation of antibiotics within the first 2 days of hospital admission.

^b^ Admission to an HDU or ICU within the first 2 days of hospital admission.

### Management and outcomes

Table 2 shows the conservative management and outcomes of the overall cohort and those stratified by antibiotic use. Antibiotics were administered to 36.2% of patients with ischaemic colitis. Of those, 94.8% were administered intravenously, and second‐ or third‐generation cephalosporins/cephamycins (78.6%; Supplemental Digital Content Table S4) were the predominant antibiotic classes. Surgeons selected antipseudomonal beta‐lactams more frequently than gastrointestinal, general medicine and general internal medicine services (12.8% vs. 5.6%). Most patients were managed with fasting (solids withheld, 97.1%) and intravenous fluids (99.7%) within the first 2 days of admission. The median [IQR] durations of fasting and intravenous fluid therapy were 3 [2–4] and 5 [4–7] days, respectively, both of which were longer in the antibiotic group. Colonoscopy was performed in 14.3% of patients within the first 2 days of hospitalization and in 35.3% at any time during hospitalization. Within the first 2 days of admission, 1.9% of patients received red blood cell transfusions.

**TABLE 2:** In‐hospital management and outcomes stratified by antibiotic use.

Management and outcomes	Total (*n* = 111750)	No antibiotic use (*n* = 71257)	Antibiotic use^a^ (*n* = 40493)	SMD
Management				
Antibiotics within the first 2 days of admission	40493 (36.2)	0 (0)	40493 (100)	N/A
Fasting within the first 2 days of admission	108542 (97.1)	69003 (96.8)	39539 (97.6)	0.05
Fasting duration, days^b^	3.0 [2.0, 4.0]	3.0 [2.0, 4.0]	3.0 [3.0, 5.0]	0.31
Intravenous fluids within the first 2 days of admission	111410 (99.7)	70958 (99.6)	40452 (99.9)	0.06
Intravenous fluids, days^b^	5.0 [4.0, 7.0]	5.0 [4.0, 7.0]	6.0 [5.0, 9.0]	0.30
Colonoscopy within the first 2 days of admission	15946 (14.3)	10481 (14.7)	5465 (13.5)	0.04
Colonoscopy during hospitalization	39464 (35.3)	24953 (35.0)	14511 (35.8)	0.02
RBC transfusion within the first 2 days of admission	2162 (1.9)	1285 (1.8)	877 (2.2)	0.03
RBC transfusion volume, units^b^	4.0 [2.0, 6.0]	4.0 [2.0, 6.0]	4.0 [2.0, 6.0]	0.10
Outcomes				
Surgery or in‐hospital death	2375 (2.1)	685 (1.0)	1690 (4.2)	0.20
Surgery	983 (0.9)	181 (0.3)	802 (2.0)	0.17
In‐hospital death	1494 (1.3)	517 (0.7)	977 (2.4)	0.14
*Clostridioides difficile* infection	220 (0.2)	59 (0.1)	161 (0.4)	0.06
Length of hospital stay	8.0 [6.0, 11.0]	8.0 [6.0, 10.0]	10.0 [7.0, 14.0]	0.32
30‐day all‐cause readmission	5091 (4.6)	2810 (3.9)	2281 (5.6)	0.08
Total hospitalization cost, US dollars^c^	3078 [2409, 4081]	2877 [2288, 3679]	3532 [2713, 4962]	0.33

*Note*: Continuous variables are presented as the median [IQR], and categorical variables as *n* (%).

Abbreviations: N/A, not applicable; RBC, red blood cell; SMD, standardized mean difference.

^a^ Antibiotic use for ischaemic colitis was defined as the initiation of antibiotics within the first 2 days of hospital admission.

^b^ Durations/volumes were calculated only for patients who received the corresponding treatment.

^c^ We converted Japanese yen to U.S. dollars at an exchange rate of approximately ¥113.2 per US$1, derived from the Bank of Japan's statistical data as the period average from April 2016 through March 2023 (https://www.stat‐search.boj.or.jp/index_en.html).

Overall, the primary outcome, that is the composite of receipt of surgery and in‐hospital mortality occurred in 2.1% (*n* = 2375) of cases; 0.9% (*n* = 983) underwent surgery and 1.3% (*n* = 1,494) died during hospitalization. The primary outcome occurred in 1.0% of cases that did not receive antibiotics and in 4.2% of cases that received antibiotics. Other outcomes were also more frequent in the antibiotic group than in the no‐antibiotic group, including higher rates of surgery (2.0% vs. 0.3%), in‐hospital death (2.4% vs. 0.7%) and *Clostridioides difficile* infection (0.4% vs. 0.1%), longer hospital stays [median (IQR) 10 (7–14) days vs. 8 (6–10) days], higher 30‐day all‐cause readmission (5.6% vs. 3.9%) and greater total hospitalization costs (¥399,860 vs. ¥325,700, approximating US$3,532 vs. US$2,877, respectively).

### Distribution of surgical approaches and procedures

**FIGURE 2:**
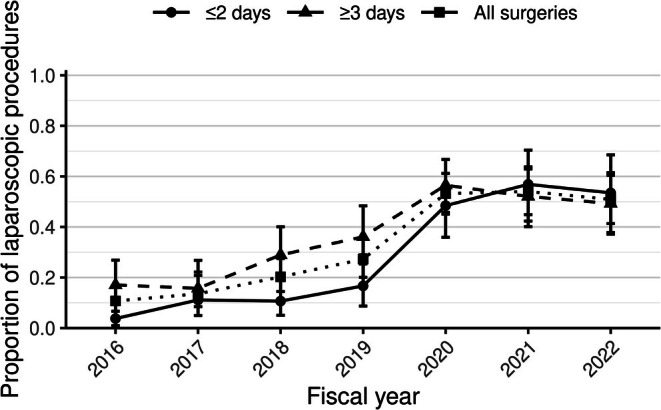
Trend in the proportion of laparoscopic surgeries by fiscal year. The annual proportions of laparoscopic surgeries among all surgeries for ischaemic colitis from fiscal year 2016 through 2022 are shown. The dotted lines denote the overall proportion among all surgeries, the solid lines denote surgeries performed within ≤2 days of hospitalization and the dashed lines represent surgeries performed on or after day 3 of hospitalization. The y‐axis indicates the proportion of laparoscopic surgeries, and the error bars represent the 95% binomial confidence intervals.

Table 3 presents the distribution of surgical approaches and procedures. Open surgeries accounted for 80.5% of the surgical approaches, while the proportion of laparoscopic surgeries rose dramatically from 10.7% in 2016 to 50.9% in 2022 (Figure 2). The upward trend in laparoscopy was evident for both early operations (within the first 2 days of hospitalization) and those performed on or after day 3 of hospitalization. Segmental colectomy or hemicolectomy (45.5% of all recorded surgical procedures) was the most frequent procedure, followed by stoma creation (38.8%) and total or subtotal colectomy (17.6%).

**TABLE 3:** Distribution of surgical approaches and procedures.

Type of surgery	No.^a^ (%)
Overall	983 (100)
Surgical approach	
Laparotomy	791 (80.5)
Laparoscopic approach	311 (31.6)
Surgical procedure	
Segmental colectomy or hemicolectomy	447 (45.5)
Total or subtotal colectomy	173 (17.6)
Proctocolectomy	90 (9.2)
Stoma creation	381 (38.8)
Abdominal exploration	95 (9.7)
Surgery for panperitonitis	39 (4.0)
Surgery for intra‐abdominal abscess	6 (0.6)

^a^ The counts indicate the number of operations performed during the entire hospitalization. The percentages indicate the proportion among cases in which surgery was performed. Because a single operation may involve more than one procedure code (e.g. segmental colectomy plus stoma formation) and patients may undergo more than one operation during the hospitalization, the totals across surgical approaches and surgical procedures may exceed the number of patients who underwent surgery.

### Multivariate analysis of the association between antibiotic use and the outcomes

Of the 111,750 eligible cases, 1,877 were excluded (1,455 were discharged and 436 underwent surgery within 2 days of admission), leaving 109,873 cases for the analysis. In the univariate models with antibiotic use as the sole predictor, antibiotic use was associated with higher odds of the composite outcome of surgery or in‐hospital death (OR 3.43, 95% CI 3.09–3.80), surgery alone (OR 3.61, 95% CI 3.02–4.32) and in‐hospital death (OR 3.34, 95% CI 2.95–3.78) (Table 4). After multivariate adjustment, these associations were attenuated, with an OR of 1.98 (95% CI 1.77–2.21) for the composite outcome, 1.95 (95% CI 1.61–2.36) for surgery alone and 1.89 (95% CI 1.65–2.15) for in‐hospital mortality. The ORs for the individual covariates are presented in Supplemental Digital Content Table S5.

**TABLE 4:** Comparison of outcomes between the no‐antibiotic and antibiotic groups.

Outcomes	No. (%)^a^		Odds ratio (95% CI)^b^	
	No antibiotic use (*n* = 70,253)	Antibiotic use (*n* = 39,620)	Unadjusted	Multivariate adjusted^c^
Surgery or in‐hospital mortality	495 (0.8)	932 (2.6)	3.43 (3.09–3.80)	1.98 (1.77–2.21)
Surgery	175 (0.3)	347 (1.0)	3.61 (3.02–4.32)	1.95 (1.61–2.36)
In‐hospital mortality	331 (0.5)	616 (1.7)	3.34 (2.95–3.78)	1.89 (1.65–2.15)

Abbreviations: CI, confidence interval.

^a^ To avoid immortal time bias and reverse causality, patients who underwent surgery or were discharged within the first 2 days of admission were excluded from this analysis. Of the 111,750 eligible records, 1,877 were excluded (1,455 were discharged and 436 underwent surgeries within 2 days of admission), leaving 109,873 records for analysis.

^b^ Reference: no‐antibiotic group.

^c^ The analysis was adjusted for age, sex, smoking status, body mass index, Barthel Index, Charlson Comorbidity Index, hypertension, dyslipidaemia, diabetes mellitus, atrial fibrillation, heart failure, coronary artery disease, cerebrovascular disease, peripheral artery disease, chronic obstructive pulmonary disease, chronic kidney disease, chronic liver disease, haematological malignancy, solid cancer, connective tissue disease, constipation, fiscal year of admission, admitting department, intensive care unit admission, high‐dependency care unit admission, teaching hospital status, bowel rest at admission, colonoscopy at admission, red blood cell transfusion at admission and rehydration therapy at admission.

### Sensitivity analysis

The baseline characteristics and outcomes of the sensitivity analysis restricted to first admissions for ischaemic colitis are shown in Supplemental Digital Content Tables S6 and S7. The total number of eligible cases decreased from 111,750 to 105,621. The results were similar to those of the main analysis: after multivariable adjustment, the OR was 2.01 (95% CI, 1.79–2.25) for the composite outcome, 2.00 (95% CI, 1.64–2.44) for surgery and 1.92 (95% CI, 1.67–2.20) for in‐hospital mortality (Supplemental Digital Content Table S7).

## DISCUSSION

To the best of our knowledge, this is the largest cohort study on ischaemic colitis, providing a comprehensive description of patient characteristics, management and outcomes in routine clinical practice. The overall outcomes appeared favourable: only 0.9% of cases required surgery, and 1.3% died in the hospital; both rates were lower than those reported by previous studies [2, 4, 18, 19]. Most cases were managed with fasting and intravenous fluids with median durations of 3 and 5 days, respectively. The predominant surgical approach shifted from open surgery to laparoscopic surgery. Antibiotics were administered to 36.2% of patients. In the unadjusted analyses, antibiotic use was strongly associated with surgery or in‐hospital mortality; after multivariable adjustment for 45 covariates, this association was attenuated but remained statistically significant.

In this study, we described the real‐world management patterns for ischaemic colitis in Japan. The 36.2% antibiotic administration rate observed in our study was lower than the 46–70% reported in other countries [8, 11, 20]. This difference may reflect differences in disease severity cases among the study populations and cross‐country practice variation. There is no established antibiotic regimen for ischaemic colitis, although broad‐spectrum antibiotics are commonly recommended [5]. While second−/third‐generation cephalosporins and cephamycins (78.6%) were the most prescribed class, surgeons were more likely to select broad‐spectrum agents such as antipseudomonal beta‐lactams, likely reflecting confounding by indication, as surgical services managed more complex patients. Moreover, a previous study suggested that physicians' perceptions of ischaemic colonic disease differ between medical and surgical specialties [21], and surgeons may engage in defensive antibiotic prescription due to their circumstances [22]. Future studies should quantify the appropriateness of antibiotic prescription for ischaemic colitis, including indications, spectrum, duration and de‐escalation.

Besides antibiotics, other conservative treatments such as bowel rest and intravenous fluid administration were nearly universal practices. Recent studies have reported a median fasting duration of approximately 4 days and a length of hospital stay of 7–9 days [8, 23], which are broadly consistent with the results of the current study (median fasting duration, 3 days; hospital stay, 8 days). Intravenous fluid administration practices are seldom described in previous studies; however, given the median duration of intravenous fluid administration of 5 days in our study, clinicians appeared to continue intravenous fluids for approximately 2 days after the patient resumed oral intake. The longer duration of fluid administration relative to fasting may reflect the gradual advancement of oral intake, a common practice in Japan. Although bowel rest and fluid replacement may support intestinal recovery, patients with ischaemic colitis are generally elderly (median age, 74 years), and prolonged fasting may increase the risk of oral frailty. The optimal durations of supportive therapies, as well as the criteria for diet advancement and discharge, warrant further evaluation.

Colonoscopy was performed in 14.3% of cases within the first 2 days of hospitalization and in 35.3% of cases during hospitalization, suggesting that the diagnoses of ischaemic colitis were made clinically and radiographically in the majority of cases. This aligns with the ACG guideline [5], which does not recommend screening colonoscopy during the acute phase in order to avoid additional bowel injury caused by reduced blood flow associated with luminal insufflation. Although a subset of ischaemic colitis occurs in the setting of distal obstructing lesions, including colorectal cancer, the need and optimal timing for post‐acute colonoscopy are uncertain [24, 25]. Because our database includes only inpatient records, we could not assess post‐discharge colorectal cancer screening or incident cancer, which is an important topic for future research.

Using a national inpatient database, our study provided representative outcome estimates for patients admitted with ischaemic colitis in Japan. The rates of surgery (0.9%) and in‐hospital mortality (1.3%) were substantially lower than the corresponding rates of 25% and 14.9% reported in a recent systematic review [4]. These concomitantly low rates likely reflect the strong association between the need for surgery and mortality [2, 18]. Because many previous studies incorporated confirmed histopathological and surgical findings in their inclusion criteria [4], their patient populations may have been biased toward more severe disease, complicating inference about the real‐world prognosis of ischaemic colitis across the full spectrum of disease severity. Among patients who underwent surgery, surgeons increasingly adopted the laparoscopic approach over time, even for early surgeries. Recent observational studies have suggested the potential benefit of emergent laparoscopic colectomy in ischaemic colitis, such as lower mortality and fewer surgical site complications compared with open colectomy, although these findings remain controversial given the high likelihood of residual confounding by indication [26, 27]. Whether this shift towards laparoscopy translates into improved outcomes warrants confirmation via future studies.

In the unadjusted analyses, antibiotic use was strongly associated with the composite of surgery and in‐hospital death. Although these associations were attenuated after covariate adjustment, their significance persisted. While we adjusted for a broad set of baseline characteristics and early treatments/procedures as indirect markers of severity, we lacked data on direct measures of disease severity, such as symptoms, physical findings, vital signs, laboratory values and radiographic features. Therefore, the possibility of residual confounding cannot be eliminated, and the results should be interpreted with caution. Nevertheless, our findings underscore the need for more rigorous evaluation of the appropriateness of antibiotic administration for ischaemic colitis, given their potential harms including *Clostridioides difficile* infection, antimicrobial resistance and added costs [28].

The limitations of this study should be acknowledged. First, detailed clinical severity data (medical history, laboratory results, distribution and extent of colitis, imaging findings) were unavailable, which may have resulted in residual confounding despite extensive adjustment. Second, our case and outcome definitions were not validated. To enhance specificity, we set concordant ICD‐10 and Japanese standard disease codes, as well as the performance of CT within 2 days of admission, as the inclusion criteria. Although the records of ischaemic colitis in the DPC database have not been validated, validation studies for other gastrointestinal conditions have shown high accuracy for the DPC records [29]. The requirement of CT could have introduced selection or referral bias; however, since CT availability is widespread in Japan, such bias is likely small. Third, the clinical indications for antibiotics and surgery were not directly observable. We attributed therapies initiated within the first 2 days of hospital admission to ischaemic colitis, but some misclassification is possible. Fourth, although our database covers >1,200 hospitals in Japan, the generalizability of our findings to other countries may be limited.

In summary, in this nationwide cohort, the short‐term outcomes of ischaemic colitis were found to be generally favourable with an in‐hospital mortality rate of 0.9% and surgery rate of 1.3%. Antibiotics were prescribed to about one‐third of patients but were not associated with improved short‐term outcomes after adjustment. Further observational studies with standardized severity assessment or randomized controlled trials are needed to investigate the effectiveness of antibiotics in ischaemic colitis.

## AUTHOR CONTRIBUTIONS

Yasuhiro Kano—planning and conducting the study, collecting and interpreting data, drafting the manuscript, final approval. Yuya Kimura—planning and conducting the study, collecting and interpreting data, critical revision of the manuscript, final approval. Hiroki Matsui—data curation, critical revision of the manuscript, final approval. Kiyohide Fushimi—collecting data, critical revision of the manuscript, final approval. Hideo Yasunaga—planning the study, critical revision of the manuscript, final approval.

## FUNDING INFORMATION

This work was supported by grants conferred by the Ministry of Health, Labour and Welfare of Japan (23AA2003 and 24AA2006).

## CONFLICT OF INTEREST STATEMENT

The authors have no conflict of interest to declare.

## ETHICS STATEMENT

This study was approved by the Institutional Review Board of the University of Tokyo (3501–5, May 19, 2021), which waived the need for informed consent due to the anonymous nature of the data source.

## Supporting information


Data S1.
**Table S1.** Definitions of comorbidities.
**TABLE S2**. Description of intravenous fluid therapy agents.
**TABLE S3**. Comparison of patient characteristics at admission by managing service (GI/GM/GIM vs. surgery).
**TABLE S4**. Distribution of antibiotic classes among patients with ischaemic colitis who received antibiotics, stratified by managing service (GI/GM/GIM vs. surgery).
**TABLE S5**. Multivariate analysis of antibiotic use and potential confounders for the composite of in‐hospital death and surgery.
**TABLE S6**. Patient characteristics stratified by antibiotic use in the sensitivity analysis restricted to first admissions for ischaemic colitis.
**TABLE S7**. Comparison of outcomes between the no‐antibiotic and antibiotic groups in the sensitivity analysis restricted to first admissions for ischaemic colitis.

## Data Availability

Research data are not shared.
