# Supplementary material for: Patient characteristics, antibiotic use, and in‐hospital outcomes in patients with ischaemic colitis: A nationwide retrospective cohort study
Source: Colorectal Dis. 2026 Feb 8;28(2):e70385. doi: 10.1111/codi.70385 (PMC12883592; doi:10.1111/codi.70385)
Supplement: Supplementary file 1 — Data S1. Table S1. Definitions of comorbidities. TABLE S2. Description of intravenous fluid therapy agents. TABLE S3. Comparison of patient characteristics at admission by managing service (GI/GM/GIM vs. surgery). TABLE S4. Distribution of antibiotic classes among patients with ischaemic colitis who received antibiotics, stratified by managing service (GI/GM/GIM vs. surgery). TABLE S5. Multivariate analysis of antibiotic use and potential confounders for the composite of in‐hospital death and surgery. TABLE S6. Patient characteristics stratified by antibiotic use in the sensitivity analysis restricted to first admissions for ischaemic colitis. TABLE S7. Comparison of outcomes between the no‐antibiotic and antibiotic groups in the sensitivity analysis restricted to first admissions for ischaemic colitis. [file CODI-28-0-s001.docx]

**Supplementary Table 1.** Definitions of comorbidities

| Comorbidity | ICD-10 codes |
| --- | --- |
| Hypertension | I10, I11.x–I13.x, I15.x |
| Dyslipidemia | E78.x |
| Diabetes mellitus | E10.x–E14.x |
| Atrial fibrillation | I48.x |
| Heart failure | I50.x |
| Coronary artery disease | I20.x–25.x |
| Cerebrovascular disease | I60.x–I69.x |
| Peripheral artery disease | I70.x, I74.x |
| Chronic obstructive pulmonary disease | J43.x, J44.x |
| Chronic kidney disease | N18.x, N19.x |
| Chronic liver disease | B18.x, I85.x, I86.4, I98.2, I98.3, K70.x, K71.x, K73.x, K74.x, K76.6, K76.7 |
| Hematologic malignancy | C81.x–C96.x |
| Solid cancer | C00.x–C26.x, C30.x–C41.x, C43.x–C58.x, C60.x–C80.x, D00.x–D09.x |
| Connective tissue disease | M05.x–M09.x, M30.x–M35.x |
| Constipation | K59.0 |

Abbreviations: ICD-10, International Classification of Diseases–10^th^ Revision

**Supplementary Table 2.** Description of intravenous fluid therapy agents

| Therapeutic category | 4-digit category code (KEGG DRUG*) |
| --- | --- |
| Glucose solutions | 3231 |
| Maltose solutions | 3233 |
| Xylitol solutions | 3234 |
| Other saccharide solutions | 3239 |
| Compound amino acid solutions | 3253 |
| Other protein/amino acid preparations | 3259 |
| Physiological saline solutions | 3311 |
| Dextran preparations | 3312 |
| Other plasma substitutes | 3319 |

* KEGG DRUG is an integrated database that consolidates drug information from Japan, the United States, and Europe based on chemical structure and active ingredients. We identified therapeutic categories using the 4-digit therapeutic classification code provided in the database.

**Supplementary Table 3.** Comparison of patient characteristics at admission by managing service (GI/GM/GIM vs surgery)

| Variables | GI/GM/GIM  (n=92545) | Surgery  (n=13907) | SMD |
| --- | --- | --- | --- |
| Age, years | 74.0 [63.0, 83.0] | 75.0 [65.0, 83.0] | 0.08 |
| Female | 69243 (74.8) | 9523 (68.5) | 0.14 |
| BMI, kg/m^2^ |  |  | 0.05 |
| <25 | 67379 (72.8) | 10419 (74.9) |  |
| ≥25 | 18142 (19.6) | 2569 (18.5) |  |
| Missing data | 7024 (7.6) | 919 (6.6) |  |
| Smoking history |  |  | 0.05 |
| Non-smoker | 67764 (73.2) | 9883 (71.1) |  |
| Current or former smoker | 16075 (17.4) | 2697 (19.4) |  |
| Missing data | 8706 (9.4) | 1327 (9.5) |  |
| Barthel Index on admission |  |  | 0.125 |
| 100 | 55787 (60.3) | 8017 (57.6) |  |
| 55–95 | 18821 (20.3) | 2532 (18.2) |  |
| 5–50 | 11435 (12.4) | 2010 (14.5) |  |
| 0 | 6407 (6.9) | 1337 (9.6) |  |
| Missing data | 95 (0.1) | 11 (0.1) |  |
| Charlson Comorbidity Index |  |  | 0.20 |
| 0 | 71836 (77.6) | 9784 (70.4) |  |
| 1 | 5772 (6.2) | 764 (5.5) |  |
| 2 | 11531 (12.5) | 2477 (17.8) |  |
| ≥3 | 3406 (3.7) | 882 (6.3) |  |
| Comorbidities |  |  |  |
| Hypertension | 25728 (27.8) | 4144 (29.8) | 0.04 |
| Dyslipidemia | 13233 (14.3) | 1862 (13.4) | 0.03 |
| Diabetes mellitus | 11508 (12.4) | 1812 (13.0) | 0.02 |
| Atrial fibrillation | 2302 (2.5) | 385 (2.8) | 0.02 |
| Congestive heart failure | 3669 (4.0) | 605 (4.4) | 0.02 |
| Ischemic heart disease | 6423 (6.9) | 992 (7.1) | 0.01 |
| Cerebrovascular disease | 5746 (6.2) | 1034 (7.4) | 0.05 |
| Peripheral artery disease | 365 (0.4) | 85 (0.6) | 0.03 |
| COPD | 661 (0.7) | 129 (0.9) | 0.02 |
| Chronic kidney disease | 2774 (3.0) | 443 (3.2) | 0.01 |
| Chronic liver disease | 1414 (1.5) | 177 (1.3) | 0.02 |
| Hematologic malignancy | 303 (0.3) | 30 (0.2) | 0.02 |
| Solid cancer | 10710 (11.6) | 2241 (16.1) | 0.13 |
| Connective tissue disease | 708 (0.8) | 80 (0.6) | 0.02 |
| Constipation | 11765 (12.7) | 1579 (11.4) | 0.04 |
| HDU admission* | 469 (0.5) | 227 (1.6) | 0.11 |
| ICU admission* | 122 (0.1) | 229 (1.6) | 0.16 |
| Teaching hospital admission | 78145 (84.4) | 8606 (61.9) | 0.53 |
| Fiscal year |  |  | 0.05 |
| 2016 | 13774 (14.9) | 2054 (14.8) |  |
| 2017 | 13729 (14.8) | 1993 (14.3) |  |
| 2018 | 13977 (15.1) | 2325 (16.7) |  |
| 2019 | 13532 (14.6) | 1998 (14.4) |  |
| 2020 | 13958 (15.1) | 2122 (15.3) |  |
| 2021 | 12684 (13.7) | 1880 (13.5) |  |
| 2022 | 10891 (11.8) | 1535 (11.0) |  |

Continuous variables are presented as the median [IQR], and categorical variables as n (%).

Abbreviations: GI, gastroenterology; GIM, general internal medicine; GM, general medicine; HDU, high-dependency care unit; ICU, intensive care unit; IQR, inter-quartile range; SD, standard deviation; SMD, standard mean difference

* Admission to an HDU or ICU within the first 2 days of hospital admission

**Supplementary Table 4.** Distribution of antibiotic classes among patients with ischemic colitis who received antibiotics, stratified by managing service (GI/GM/GIM vs surgery)

| Types of antibiotics | Total*  n= 40493 | GI/GM/GIM  n= 31025 | Surgery  n= 7195 |
| --- | --- | --- | --- |
| Intravenous | 38405 (94.8%) | 29199 (94.1%) | 7027 (97.7%) |
| Oral | 2664 (6.6%) | 2275 (7.3%) | 257 (3.6%) |
| Second-and third-generation cephalosporins/cephamycins | 31842 (78.6%) | 24497 (79.0%) | 5742 (79.8%) |
| Fluoroquinolones | 1962 (4.8%) | 1693 (5.5%) | 165 (2.3%) |
| Ampicillin/sulbactam | 1782 (4.4%) | 1401 (4.5%) | 218 (3.0%) |
| Antipseudomonal beta-lactams | 2997 (7.4%) | 1736 (5.6%) | 920 (12.8%) |
| Piperacillin–tazobactam | 1471 (3.6%) | 56 (0.2%) | 31 (0.4%) |
| Fourth-generation cephalosporins | 185 (0.5%) | 130 (0.4%) | 34 (0.5%) |
| Carbapenems | 1370 (3.4%) | 731 (2.4%) | 482 (6.7%) |
| Monobactams | 2 (0.0%) | 0 (0.0%) | 1 (0.0%) |
| Metronidazole | 262 (0.6%) | 170 (0.5%) | 61 (0.8%) |
| Anti-MRSA antibiotics | 128 (0.3%) | 57 (0.2%) | 40 (0.6%) |
| Glycopeptides | 124 (0.3%) | 56 (0.2%) | 38 (0.5%) |
| Daptomycin | 2 (0.0%) | 1 (0.0%) | 1 (0.0%) |
| Linezolid | 2 (0.0%) | 0 (0.0%) | 1 (0.0%) |

Counts are given as the number of patients (%), as individuals may have received more than one class of antibiotic, categories are non-exclusive and column percentages may add to >100%.

Abbreviations: GI, gastrointestinal; GIM, general internal medicine; GM, general medicine; GI, gastrointestinal; MRSA, methicillin-resistant *Staphylococcus aureus*

* The total includes patients managed by GI/GM/GIM, surgery, and other services.

**Supplementary Table 5.** Multivariate analysis of antibiotic use and potential confounders for the composite of in-hospital death and surgery

| Variables | Multivariate-adjusted  odds ratio (95% CI)* |
| --- | --- |
| Age^†^ | 1.03 (1.02–1.03) |
| Sex |  |
| Men | Reference |
| Women | 0.62 (0.55–0.70) |
| Body mass index (kg/m^2^) |  |
| <25 | Reference |
| ≥25 | 0.77 (0.65–0.91) |
| Missing data | 1.12 (0.94–1.32) |
| Smoking status |  |
| Non-smoker | Reference |
| Current/former smoker | 1.18 (1.02–1.36) |
| Missing data | 1.03 (0.87–1.22) |
| Barthel Index on admission |  |
| 100 | Reference |
| 55–95 | 1.30 (1.08–1.56) |
| 5–50 | 2.72 (2.31–3.21) |
| 0 | 6.53 (5.56–7.68) |
| Missing data | 1.13 (0.06–5.24) |
| Charlson comorbidity index |  |
| 0 | Reference |
| 1 | 1.51 (1.22–1.86) |
| 2 | 2.06 (1.78–2.37) |
| ≥3 | 2.99 (2.42–3.68) |
| Hypertension |  |
| No | Reference |
| Yes | 0.68 (0.60–0.77) |
| Dyslipidemia |  |
| No | Reference |
| Yes | 0.71 (0.59–0.86) |
| Diabetes mellitus |  |
| No | Reference |
| Yes | 1.03 (0.90–1.18) |
| Atrial fibrillation |  |
| No | Reference |
| Yes | 1.51 (1.23–1.85) |
| Heart failure |  |
| No | Reference |
| Yes | 1.02 (0.85–1.23) |
| Coronary artery disease |  |
| No | Reference |
| Yes | 1.14 (0.96–1.35) |
| Cerebrovascular disease |  |
| No | Reference |
| Yes | 1.00 (0.85–1.17) |
| Peripheral artery disease |  |
| No | Reference |
| Yes | 1.62 (1.01–2.49) |
| COPD |  |
| No | Reference |
| Yes | 1.66 (1.18–2.30) |
| Chronic kidney disease |  |
| No | Reference |
| Yes | 2.05 (1.70–2.47) |
| Chronic liver disease |  |
| No | Reference |
| Yes | 1.09 (0.77–1.51) |
| Hematologic malignancy |  |
| No | Reference |
| Yes | 0.38 (0.13–0.85) |
| Solid cancer |  |
| No | Reference |
| Yes | 1.01 (0.86–1.18) |
| Connective tissue disease |  |
| No | Reference |
| Yes | 0.88 (0.49–1.47) |
| Constipation |  |
| No | Reference |
| Yes | 0.75 (0.62–0.90) |
| Fiscal year |  |
| 2016 | Reference |
| 2017 | 0.92 (0.75–1.12) |
| 2018 | 0.99 (0.82–1.21) |
| 2019 | 0.86 (0.70–1.06) |
| 2020 | 1.10 (0.90–1.33) |
| 2021 | 1.02 (0.83–1.24) |
| 2022 | 1.18 (0.97–1.45) |
| Department |  |
| Surgery | Reference |
| Gastroenterology | 0.20 (0.18–0.23) |
| GM/GIM | 0.24 (0.21–0.28) |
| Others | 0.64 (0.54–0.76) |
| ICU admission |  |
| No | Reference |
| Yes | 3.60 (2.51–5.12) |
| HDU admission |  |
| No | Reference |
| Yes | 1.57 (1.14–2.11) |
| Teaching hospital |  |
| No | Reference |
| Yes | 0.99 (0.87–1.13) |
| Bowel rest at admission |  |
| No | Reference |
| Yes | 0.60 (0.48–0.75) |
| Colonoscopy at admission |  |
| No | Reference |
| Yes | 0.88 (0.75–1.02) |
| RBC transfusion at admission |  |
| No | Reference |
| Yes | 2.28 (1.88–2.75) |
| Rehydration therapy at admission |  |
| No | Reference |
| Yes | 0.58 (0.34–1.04) |

Abbreviations: CI, confidence interval; COPD, chronic obstructive pulmonary disease; GIM, general internal medicine; GM, general medicine; HDU, high-dependency care unit; ICU, intensive care unit; RBC, red blood cell

^†^ Age was modeled as a continuous covariate; odds ratios represent 1-year increments.

**Supplementary Table 6.** Patient characteristics stratified by antibiotic use in the sensitivity analysis restricted to first admissions for ischemic colitis

| Variables | Total  (n = 105621) | No antibiotic use  (n = 67048) | Antibiotic use*  (n = 38573) | SMD |
| --- | --- | --- | --- | --- |
| Age, years | 74.0 [63.0, 83.0] | 73.0 [61.0, 82.0] | 76.0 [66.0, 84.0] | 0.22 |
| Female | 77434 (73.3) | 50312 (75.0) | 27122 (70.3) | 0.11 |
| BMI, kg/m^2^ |  |  |  |  |
| <25 | 77100 (73.0) | 49158 (73.3) | 27942 (72.4) | 0.02 |
| ≥25 | 20492 (19.4) | 13167 (19.6) | 7325 (19.0) | 0.02 |
| Missing data | 8029 (7.6) | 4723 (7.0) | 3306 (8.6) | 0.06 |
| Smoking history |  |  |  |  |
| Non-smoker | 76525 (72.5) | 49074 (73.2) | 27451 (71.2) | 0.05 |
| Current or former smoker | 18824 (17.8) | 11843 (17.7) | 6981 (18.1) | 0.01 |
| Missing data | 10272 (9.7) | 6131 (9.1) | 4141 (10.7) | 0.05 |
| Barthel Index on admission |  |  |  |  |
| 100 | 62695 (59.4) | 42821 (63.9) | 19874 (51.5) | 0.25 |
| 55–95 | 21116 (20.0) | 13375 (19.9) | 7741 (20.1) | 0.00 |
| 5–50 | 13590 (12.9) | 7223 (10.8) | 6367 (16.5) | 0.17 |
| 0 | 8097 (7.7) | 3545 (5.3) | 4552 (11.8) | 0.23 |
| Missing data | 123 (0.1) | 84 (0.1) | 39 (0.1) | 0.01 |
| Charlson Comorbidity Index |  |  |  |  |
| 0 | 80345 (76.1) | 52884 (78.9) | 27461 (71.2) | 0.18 |
| 1 | 6614 (6.3) | 3797 (5.7) | 2817 (7.3) | 0.07 |
| 2 | 14180 (13.4) | 8006 (11.9) | 6174 (16.0) | 0.12 |
| ≥3 | 4482 (4.2) | 2361 (3.5) | 2121 (5.5) | 0.10 |
| Comorbidities |  |  |  |  |
| Hypertension | 29288 (27.7) | 17858 (26.6) | 11430 (29.6) | 0.07 |
| Dyslipidemia | 14668 (13.9) | 9302 (13.9) | 5366 (13.9) | 0.00 |
| Diabetes mellitus | 13308 (12.6) | 7452 (11.1) | 5856 (15.2) | 0.12 |
| Atrial fibrillation | 2665 (2.5) | 1543 (2.3) | 1122 (2.9) | 0.04 |
| Congestive heart failure | 4380 (4.1) | 2407 (3.6) | 1973 (5.1) | 0.08 |
| Ischemic heart disease | 7324 (6.9) | 4328 (6.5) | 2996 (7.8) | 0.05 |
| Cerebrovascular disease | 6700 (6.3) | 3808 (5.7) | 2892 (7.5) | 0.07 |
| Peripheral artery disease | 472 (0.4) | 224 (0.3) | 248 (0.6) | 0.04 |
| COPD | 806 (0.8) | 436 (0.7) | 370 (1.0) | 0.04 |
| Chronic kidney disease | 3290 (3.1) | 1721 (2.6) | 1569 (4.1) | 0.08 |
| Chronic liver disease | 1570 (1.5) | 960 (1.4) | 610 (1.6) | 0.01 |
| Hematologic malignancy | 371 (0.4) | 211 (0.3) | 160 (0.4) | 0.02 |
| Solid cancer | 13058 (12.4) | 8076 (12.0) | 4982 (12.9) | 0.03 |
| Connective tissue disease | 847 (0.8) | 487 (0.7) | 360 (0.9) | 0.02 |
| Constipation | 12902 (12.2) | 8256 (12.3) | 4646 (12.0) | 0.01 |
| HDU admission^†^ | 729 (0.7) | 241 (0.4) | 488 (1.3) | 0.10 |
| ICU admission^†^ | 410 (0.4) | 57 (0.1) | 353 (0.9) | 0.12 |
| Teaching hospital admission | 86711 (82.1) | 56085 (83.6) | 30626 (79.4) | 0.11 |
| Attending department |  |  |  |  |
| Gastroenterology | 54549 (51.6) | 36268 (54.1) | 18281 (47.4) | 0.13 |
| Surgery | 13146 (12.4) | 6286 (9.4) | 6860 (17.8) | 0.25 |
| General (Internal) Medicine | 32919 (31.2) | 21639 (32.3) | 11280 (29.2) | 0.07 |
| Other | 5007 (4.7) | 2855 (4.3) | 2152 (5.6) | 0.06 |
| Fiscal year |  |  |  |  |
| 2016 | 16261 (15.4) | 9928 (14.8) | 6333 (16.4) | 0.04 |
| 2017 | 15870 (15.0) | 9802 (14.6) | 6068 (15.7) | 0.03 |
| 2018 | 16366 (15.5) | 10289 (15.3) | 6077 (15.8) | 0.01 |
| 2019 | 15344 (14.5) | 10087 (15.0) | 5257 (13.6) | 0.04 |
| 2020 | 15703 (14.9) | 10052 (15.0) | 5651 (14.7) | 0.01 |
| 2021 | 14040 (13.3) | 9045 (13.5) | 4995 (12.9) | 0.02 |
| 2022 | 12037 (11.4) | 7845 (11.7) | 4192 (10.9) | 0.03 |

Continuous variables are presented as the median [IQR], and categorical variables as n (%).

Abbreviations: BMI, body mass index; COPD, chronic obstructive pulmonary disease; HDU, high-dependency care unit; ICU, intensive care unit; IQR, interquartile range; SD, standard deviation; SMD, standardized mean difference

*Antibiotic use for ischemic colitis was defined as initiation of antibiotics within the first 2 days of hospital admission.

^†^Admission to an HDU or ICU within the first 2 days of hospital admission.

**Supplementary Table 7.** Comparison of outcomes between the no-antibiotic and antibiotic groups in the sensitivity analysis restricted to first admissions for ischemic colitis

| Outcomes | No. (%) | | Odds ratio (95% CI)^†^ | |
| --- | --- | --- | --- | --- |
|  | No antibiotic use (n=66096) | Antibiotic use (n=37723) | Unadjusted | Multivariate adjusted^‡^ |
| Surgery or in-hospital mortality | 495 (0.8) | 932 (2.6) | 3.50 (3.14–3.90) | 2.01 (1.79–2.25) |
| Surgery | 175 (0.3) | 347 (1.0) | 3.70 (3.08–4.47) | 2.00 (1.64–2.44) |
| In-hospital mortality | 331 (0.5) | 616 (1.7) | 3.40 (2.99–3.86) | 1.92 (1.67–2.20) |

^†^Reference: no-antibiotic group

^‡^ Multivariate analysis was adjusted for age, sex, smoking status, body mass index, Barthel Index, Charlson Comorbidity Index, hypertension, dyslipidemia, diabetes mellitus, atrial fibrillation, heart failure, coronary artery disease, cerebrovascular disease, peripheral artery disease, chronic obstructive pulmonary disease, chronic kidney disease, chronic liver disease, hematological malignancy, solid cancer, connective tissue disease, constipation, fiscal year of admission, admitting department, intensive care unit admission, high-dependency care unit admission, teaching hospital status, bowel rest at admission, colonoscopy at admission, red blood cell transfusion at admission, and rehydration therapy at admission.

Abbreviations: CI, confidence interval
